# A Review: Research Progress of Neural Probes for Brain Research and Brain–Computer Interface

**DOI:** 10.3390/bios12121167

**Published:** 2022-12-14

**Authors:** Jiahui Luo, Ning Xue, Jiamin Chen

**Affiliations:** 1 State Key Laboratory of Transducer Technology, Aerospace Information Research Institute, Chinese Academy of Sciences, Beijing 100190, China; 2 School of Electronic, Electrical and Communication Engineering, University of Chinese Academy of Sciences, Beijing 100049, China

**Keywords:** micromechanical technology, neural probes, electrodes flexibility, optogenetics, magnetic recordings, brain–computer interface

## Abstract

Neural probes, as an invasive physiological tool at the mesoscopic scale, can decipher the code of brain connections and communications from the cellular or even molecular level, and realize information fusion between the human body and external machines. In addition to traditional electrodes, two new types of neural probes have been developed in recent years: optoprobes based on optogenetics and magnetrodes that record neural magnetic signals. In this review, we give a comprehensive overview of these three kinds of neural probes. We firstly discuss the development of microelectrodes and strategies for their flexibility, which is mainly represented by the selection of flexible substrates and new electrode materials. Subsequently, the concept of optogenetics is introduced, followed by the review of several novel structures of optoprobes, which are divided into multifunctional optoprobes integrated with microfluidic channels, artifact-free optoprobes, three-dimensional drivable optoprobes, and flexible optoprobes. At last, we introduce the fundamental perspectives of magnetoresistive (MR) sensors and then review the research progress of magnetrodes based on it.

## 1. Introduction

The neural network of the human brain is composed of an estimated 86 billion neurons [1], which convey information across complex temporal patterns within the neuronal network. Deciphering the fundamental mechanisms and processes of the human mind plays an important role in recognizing human thoughts, emotions, and ultimately explains human behavior. On the other hand, the brain–computer interface (BCI), which allows direct communication between the brain’s electrical activity and an external device [2], most commonly a computer, does not rely on conventional brain information output pathways (peripheral nerve and muscle tissue) and has been widely concerned in medical, industrial, and household settings [3,4]. In order to realize the bidirectional interaction, it is essential for accurate acquisition and feedback of neural signals.

Activities in support of the deciphering of nervous system codes and the bidirectional interaction of BCIs rely on advanced methodologies and engineering systems at different scales [5]. Macroscopically, non-invasive methods such as electroencephalogram (EEG) [6,7,8,9], functional near-infrared spectroscopy (fNIRS) [10,11], and magnetoencephalography (MEG) [12,13,14,15] have been widely utilized due to their high time resolution. However, to analyze the neural circuits behind cognition and behavior, it is also necessary to understand how neurons connect and communicate at the cellular and molecular levels; therefore, the ideal sensing tool must span from the single neuron to its complex network of connections [16]. Neural probes are defined as devices inserted or implanted into the brain or other nervous tissues [17] that meet the above requirements.

Electrophysiological neural probes already have mature tools at different scales; patch clamps, which can record electrical activities at a single-cell scale, are the best tool for studying ion channel activity. With the development of microelectromechanical systems (MEMS) technology, high-density Si-based microelectrode arrays (MEAs) have successfully realized the recording of high-throughput and high time resolution of brain electrical signals. However, as the size of the electrode recording site decreases, it leads to low capacitance and high impedance at the electrode/tissue interface, which seriously affects the recording resolution [18,19]. At the same time, the Young’s modulus mismatch between traditional rigid electrode materials and soft biological tissues exacerbates the rejection of the probe invasive site, resulting in a decrease in electrode performance. Therefore, recent studies are more interested in the flexibility of neural microelectrodes, as it is expected to increase the charge storage capacity and reduce the interfacial impedance, thereby improving the signal-to-noise ratio (SNR) of electrophysiological signal detection.

In addition, many state-of-the-art neural probes for brain research have been reported over the past few decades. Among these achievements, the genetic modification of nerve cells with ion channels that are sensitive to light brought the promising new method of “optogenetics” into the neurosciences [20] with millisecond temporal resolution and single-cell spatial resolution [21]. The optoprobes embodied the theory of optogenetics through engineering design, providing a powerful tool for neuroscience research. In addition, magnetophysiology, concentrating on the magnetic field signals generated by ionic neuronal currents according to Biot–Savart law, acts as a complementary technique to electrical measurements with the advantages of non-contact, non-distortion, and no reference. The neural probe for magnetophysiology technology is to integrate micron-size magnetoresistance (MR) sensors based on spin electronics on a needle-shaped micromachined probe, named “magnetrodes” [22] in this review for a magnetic equivalent of electrodes. Intrusive magnetic recording of magnetrodes allows the distance from the field source to sensors to be shortened, so the amplitude of the signal (expected to be no larger than a few nT) is larger than that of MEG (hundreds of fT).

Herein, the strategy of electrode flexibility will be discussed first, including the selection of flexible substrates and new electrode materials. In the following section, the concept of optogenetics will be introduced, followed by the review of several novel structures of optoprobes, which are divided into multifunctional optoprobes integrated with microfluidic channels, artifact-free optoprobes, three-dimensional drivable optoprobes, and flexible optoprobes. In Section 4, we will introduce the basics of several types of MR effects including giant magnetoresistance (GMR) and tunneling magnetoresistance (TMR). The research progress of magnetrodes based on MR sensors will be reviewed in the same section. Finally, we provide an outlook on the current problems and future development directions of these novel neural probes. It is expected that a comprehensive and up-to-date review of the novel neural probes can be achieved.

## 2. Microelectrodes

### 2.1. Rigid Microelectrodes

As one of the most mature tools for neural probes, implantable microelectrodes can accurately record electrical signals at the neuron level. For this reason, they have been widely applied to the basis of neurobiology, and have greatly promoted the development of BCI. According to the different electrode materials, microelectrodes can be divided into glass micropipette electrodes [23,24,25], metal microwire electrodes [26,27,28], and semiconductor substrate electrodes [29,30,31]. Glass micropipette electrodes, also known as patch clamps, are used to record the electrical activity of ion channels on biological membranes [32]. Compared with micropipette electrodes made of high-temperature drawn capillary glass tubes, microwire electrodes have lower high-frequency impedance, higher signal-to-noise ratio, and better mechanical properties, which can detect the fluctuation of voltage value without damaging cell activity. Microwire electrodes are the earliest implantable microelectrodes used for long-term recording of brain activity [33]. However, when the number of channels increases, the distance between the microwire electrodes cannot be precisely controlled during arrangement so that the consistency of electrode performance cannot be guaranteed, and electrode arrays assembly is also not easy to achieve [34].

With the development of photolithography and silicon etching technology, metal microwire microelectrodes are gradually replaced by silicon microelectrodes with good mechanical properties and biocompatibility. The Utah electrode [35,36] and Michigan electrode [37] are the two most representative types of silicon-based microelectrode arrays. For the Utah electrode array (UEA), the electrode recording point is exposed at the tip of each microneedle by mechanical cutting combined with chemical etching, and then metal is deposited [36]. The rest of the needle is insulated with polyimide to obtain a microelectrode array with precise size and spacing [38]. UEA-based BCI systems have been approved by the United States Food and Drug Administration (FDA) for some clinical trials. In 2006, Hochberg et al. [39] used implantable BCI for the first time to enable quadriplegic patients to drive computer screen cursors and activate simple robotic devices just by thinking. Over the years, the number of clinical studies and the leaps they have made in the clinical field have increased significantly. In March of this year, Chaudhary et al. [40] implanted BCI into the amyotrophic lateral sclerosis (ALS) patient who lost all muscle-based communication pathways and he selected a letter to form words and phrases to communicate his needs and experiences via auditory neurofeedback training, which also proved that brain-based volitional communication is possible even in a completely locked-in state. The UEA is not only used for recording, but also for stimulation purposes. For example, inducing tactile feedback in the hand region of the somatosensory cortex may help improve the accuracy of BCI devices [41], as well as the dexterity of prosthetics [42].

The Michigan electrode array is a needle electrode similar to UEA. The difference is that it has multiple plane recording sites on the needle [43,44,45], which can achieve high-density stereo recording. In 2017, Barz et al. [46] chronically implanted assembled 3D Michigan electrode arrays into non-human primates trained to perform a reach and grasp motor task. This result supports the design of application-specific neural interfaces in neuroscience research.

### 2.2. Strategies for Microelectrode Flexibility

Based on MEMS technology, microelectrode arrays made of rigid silicon can effectively obtain high-density activity information of brain neurons. Nevertheless, the rigidity of silicon makes it unable to match the physical properties of biological tissues. At the same time, because of its non-deformable characteristics, it will cause damage to cells when the tissue moves, so it is not suitable for long-term implantation in the human body. Therefore, it is desirable to optimize the performance of implantable electrodes through electrode flexibility.

**Figure 1:**
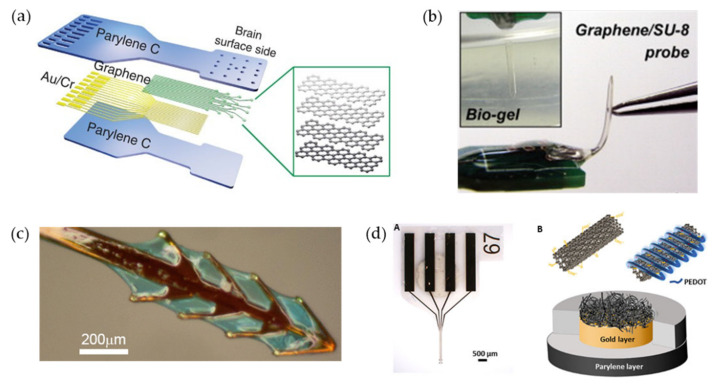
Different strategies for electrode flexibility. (**a**) Schematic of the electrode with flexible Parylene probes [52]. Reprinted under a Creative Commons Attribution (CC BY) license. (**b**) Optical micrographs of the flexible SU-8 probe with 90° bending and penetrate an agar gel. Reprinted from [54], Copyright (2013), with permission from Elsevier. (**c**) Microscope image of the fishbone-shaped polyimide neural probe [55]. (**d**) Nanomaterials PEDOT-CNF to improve electrode performance: A—Optical micrograph of the neural probe; B—Schematic diagram of the PEDOT-CNF composite deposition [66]. Reprinted under a Creative Commons Attribution (CC BY) license.

The flexibility of neural electrodes includes choosing flexible polymers as substrates and replacing traditional metal electrodes with various new electrode materials. Firstly, an electrode with flexible probes is a typical method for electrode flexibility, providing an interface more suitable for neural tissue by exhibiting more suitability (Figure 1a,b). The selected flexible substrate materials should have good biocompatibility, flexibility, and compatibility with the microfabrication processes, such as polydimethylsiloxane (PDMS) [47,48,49], polyimide (PI) [50,51], Parylene [52,53], and SU-8 [54]. Additionally, the geometry of the probe can also affect the rejection of the local tissue near the electrode. As shown in Figure 1c, Wu et al. designed a fishbone-shaped polyimide neural electrode that effectively reduced the tissue reaction by increasing the distance between the electrode and the probe [55].

On the other hand, electrode performance can be further improved by various types of organic electroactive electrode materials with high charge injection capability and excellent electrochemical performance, which includes carbon-based nanomaterials, i.e., carbon nanotubes (CNTs) [56,57,58], graphene [59,60,61], and conductive polymers (CPs) [62,63,64]. Further, nanocomposites of the above materials have also become popular choices. For example, combining conducting polymer Poly (3,4-ethylenedioxythiophene) (PEDOT) with carbon-based nanomaterials with high mechanical hardness can prevent PEDOT films from deforming and cracking after long-term operation [65]. Recently, Vajrala et al. [66] fabricated novel nanocomposites of highly porous and robust PEDOT-CNF by a simple and reproducible electrodeposition method (Figure 1d), and the experimental results showed that it has superior performance to pure PEDOT materials.

### 2.3. Methods of Flexible Microelectrode Insertion

Some studies have confirmed that flexible electrodes can indeed reduce the impact on the surrounding brain tissue [67]. However, an important problem is that flexible neural probes may be too soft to penetrate the meninges and reach the target site, thus requiring the use of additional stiffening structures. The method of stiff backbone layers [68] and insertion shuttles [69,70,71] can improve the rigidity of flexible probes, but the former will limit the flexibility of the device, and the latter will temporarily increase the footprint of the implant, causing additional damage to the nerve tissue during the implantation process. A more acceptable approach is to temporarily reinforce the probe with a bioresorbable coating, which restores the flexibility of the probe after the coating dissolves. Commonly used bioabsorbable coatings include poly (ethylene glycol) (PEG) [72], poly (lactic-co-glycolic acid) (PLGA) [73], silk fibroin [74], sucrose [75], maltose [76], dextran [77], and their bilayer structures [78,79]. More than just an insertion aid, these polymers act as biofriendly coatings to mitigate rejection. In actual use, the appropriate bio-coating should be selected according to the application scenario, combined with the stiffness, degradation rate, and bioresorbability of the polymer [80].

## 3. Optoprobes

### 3.1. Optogenetics

Optogenetics fuses optical and genetic techniques to inject photosensitive proteins (called opsins) extracted from algae or bacteria into target tissues [81,82]. When light of a specific wavelength illuminates opsins, it causes excitatory or inhibitory activity in the neuron. Traditional electrical stimulation cannot precisely control the stimulation site and drugs or genetic mutations cannot decide when to activate, while optogenetic technology can stimulate a specific site with expected time with high selectivity, spatial and temporal resolution, and reversibility [21].

Opsins, similar to human photoreceptor cells, act as photoreceptors to be regulated by specific wavelengths of light to switch ion channels and form action potentials. A variety of opsins have been used in optogenetics. ChR2, for example, when irradiated with a 470 nm blue laser, the channels of these opsins open, allowing a large influx of cations (such as Na^+^) to generate action potentials, that is, to excite neurons [83,84]. The inhibitory opsin NpHR3, in contrast, allows Cl^−^ to pass through when irradiated with a 578 nm yellow laser light, keeping the neurons at resting potential all the time [85].

### 3.2. Optoprobes

Nevertheless, due to the absorption and scattering of light by the living body, natural light cannot be irradiated deep into the brain. In order to deliver light into the specific area that needs to be stimulated, an invasive probe which is called an optoprobe [86] was developed. According to the different integration methods of optical components, optoprobes can be divided into optical waveguide probes, MEMS waveguide integrated probes, and micro-LED (μ-LED) integrated probes.

Optical-fiber-based probes, which directly use commercially available fibers and lasers as light sources, were the first way to introduce light into animals [87]. Although mature products are available, the bulky size of the fiber and the difficult manual assembly limited the further development. Although tapered fiber can reduce the size of the fiber tip by wet etching [88], the Young’s modulus is still high [89].

Thanks to the MEMS technique, MEMS waveguide integrated probes have great flexibility in design and application. The size of the probe can be significantly reduced, taking advantage of the planar profile of the platform, and thus reduce the damage caused by implantation. At the same time, the structure defined by the photolithography process leads to a compact footprint and is easy to integrate with multiple channels and multiple functions. As with optical-fiber-based probes, lasers or integrated LEDs/LDs can be selected as light sources. Integrating an interfacial optical component such as a gradient-index (GRIN) lens to improve the coupling efficiency between the light source and the waveguide has received much attention recently [90].

Probes with integrated light sources directly implant the μ-LED light source into the body without using an optical guide structure. LEDs have been applied to the tool of optical neuromodulation due to their improved encapsulation quality and negligible heat generation [91]. Due to the substrate lift-off and transfer techniques, the size of the μ-LED is reduced, which can obviously reduce the probe volume or facilitate high-density integration.

In order to achieve closed-loop optogenetic regulation, electrodes are usually integrated on optoprobes as recording elements for electrical signals generated by optical stimulation, also known as “Optrode” [20]. Optrode enables bidirectional interaction between neural interface devices and neurons, making it an indispensable tool for optogenetics applications. Interestingly, many attractive new structures have been developed recently based on optrodes, including multifunctional optoprobes integrated with microfluidic channels, artifact-free optoprobes, three-dimensional drivable optoprobes, and flexible optoprobes. These improvements are expected to enable long-term, multimodal, multifunctional studies of brain function while minimizing damage to the brain from implantation.

#### 3.2.1. Multifunctional Optoprobes

The ability of chemical delivery is crucial for the in-depth study and precise modulation of neural circuit function; therefore, recent studies have integrated microfluidic channels on optrodes to constitute multifunctional optoprobes, respectively. Microfluidic channels can infuse a drug in the deep brain region of small animals, such as anti-inflammatory drugs, which may improve the longevity of chronic recordings. Moreover, it allows for a single-insertion implantation surgery to complete the infusion of opsins and subsequent optogenetic modulation, avoiding damage to brain tissue caused by repeated device insertion in the same region [92].

**Figure 2:**
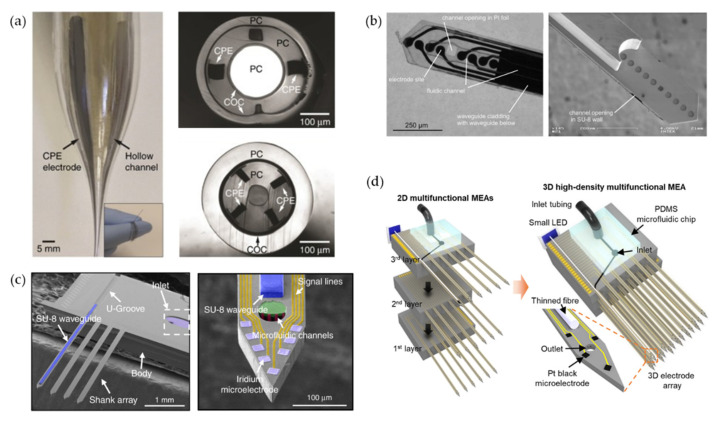
Different kinds of multifunctional optoprobes integrating optical stimulation, electrical recording, and microfluidic channels. (**a**) Multifunctional coaxial polymer fiber-based optoprobe achieved by thermal drawing process. Refractive index difference between medical-grade materials- polycarbonate (PC) and cyclic olefin copolymer (COC) allow light to be confined within PC, while the polymer composite-conductive polyethylene (CPE) is used as recording electrode [94]. (**b**) Micrographs of two multifunctional optoprobes with different waveguide output surfaces (flat, concave) which might influence the light propagation in tissue [95]. (**c**) View of the multifunctional MEMS two-dimensional multi-handle waveguide-based optoprobes. Reprinted under a Creative Commons Attribution (CC BY) license [96]. (**d**) Schematic illustrations showing three 2D multifunctional optoprobes before stacking and bonding (left), assembled 3D high-density multifunctional array (middle) [97]. Reprinted under a Creative Commons Attribution (CC BY) license.

Coaxial electrodes and microfluidic channels were fabricated on a pharmaceutical-grade polymer optical fiber via the thermal drawing process, which first realized virus infusion [93] and drug delivery (Figure 2a) [94]. The drug was infused through the void space for fluid delivery in the cylindrical fiber [91]. Rubehn et al. used the micromachining process to integrate an SU-8 waveguide, as shown in Figure 2b, and fluidic channel into a polyimide-based electrode shaft [95], but these methods all require manual assembly or bonding, resulting in poor scalability of probes. Shin et al. designed a multifunctional two-dimensional multi-handle optoprobe (Figure 2c) [96] and three-dimensional high-density array (Figure 2d) [97] capable of confirming the functional connectivity of different brain regions in mice on a silicon-based substrate for the first time.

#### 3.2.2. Artifact-Free Optoprobes

An undesirable feature of many optical devices is the stimulation artifact that may mask neuronal signals and prevent the temporally precise recording of neuronal responses [98,99]. It is showed that the magnitude of stimulation artifacts is often an order of magnitude larger than those of underlying neuronal signals [100]. Especially for μ-LED-integrated optoprobes. LED, as an active device, has an obvious impact on the electrical recording when it is switching. Optical-stimulation-induced artifacts mainly include photovoltaic effects (PV), electromagnetic interference (EMI), and photoelectrochemical effects (PEC). Comparison of the three types of optical-stimulation-induced artifacts is shown in Table 1.

Metal microelectrodes exposed to optical radiation are susceptible to the PV effect, producing stimulus-locked optoelectronic artifacts [101,102,103,104]. This artifact can be mitigated using heavily boron-doped silicon substrates [100], but still cannot be completely eliminated due to the presence of PEC noise. To minimize the effect of optoelectronic artifacts, highly transparent materials such as graphene [52] or indium tin oxide [105] have been used as electrode materials. Flexible polymer-based substrates to eliminate PV-induced artifacts are also a good option [106,107,108].

Active μ-LEDs and their interconnects will introduce EMI-induced artifacts to the electrical recording signal [107,108,109], which bring the largest amplitude of artifacts. When μ-LEDs work, due to the existence of contact resistance and lead resistance, their N-type layer cannot maintain a stable ground potential, which affects the recording point of the upper layer. Adding a metal shielding layer between the μ-LED active layer and the electrode recording passive layer can effectively reduce EMI [100,108,110]. The dual-metal-layer shielding topology has been applied in µ-LED-based photoelectrodes to reduce stimulation artifacts and has shown superior performance to the single-metal-layer topology [111]. In addition, transient pulse shaping using the LED drive signal can effectively suppress EMI noise [110].

The PEC noise on the metal–electrolyte interface can be mitigated by using electrochemical modification of materials [102,106,112,113,114] with a band gap of larger than 3.26 eV, such as counterion-doped PEDOT, Sn-doped indium oxide, or Pt-Black [52,106,115].

#### 3.2.3. Three-Dimensional Drivable Optoprobes

**Figure 3:**
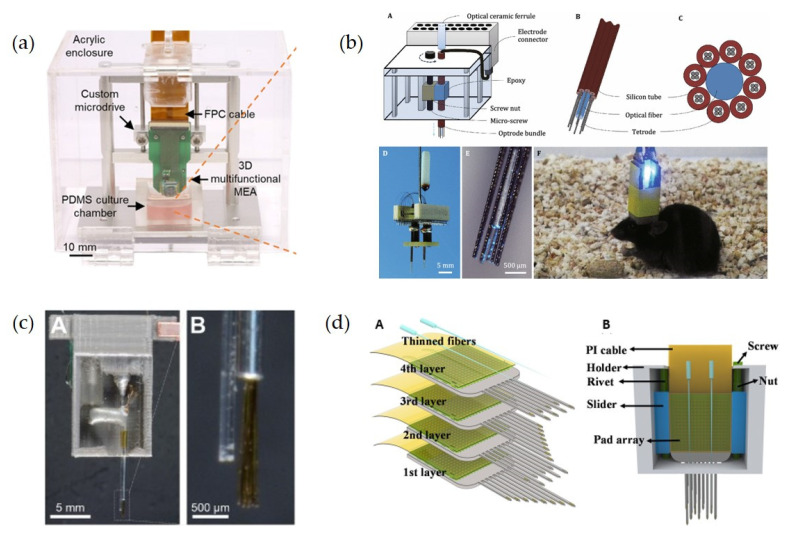
Several types of three-dimensional drivable optoprobes. (**a**) Photograph of the 3D optoprobe with a custom microdrive [97]. Reprinted under a Creative Commons Attribution (CC BY) license. (**b**) Design of multisite drivable fiber-based optrode arrays. (**A**): Schematic diagram of a 32-channel drivable optrode array; (**B**): Detailed schematic diagram and top view (**C**) of the optrode tip; (**D**): Photos of a 64-channel multisite drivable optrode array and the optrode tip (**E**); (**F**): Optogenetic stimulation and electrophysiological recording using a multisite drivable optrode array implanted in a freely moving mouse [116]. Reprinted under a Creative Commons Attribution (CC BY) license. (**c**) The fully prepared drivable optrode with the 3D-printed acrylic microdrive and magnified view of the optrode tip. (**A**): Front view of the drivable optrode; (**B**): Magnified image of the optrode tip [122]. Reprinted from, copyright (2021), with permission from Elsevier. (**d**) Schematic diagram of the completely constructed 3D high-density drivable optrode array. (**A**): Explosive view of four 2D high-density probes; (**B**): Schematic diagram of the completely constructed 3D high-density drivable optrode array. Reprinted with permission from [121]. Copyright 2021 American Chemical Society.

When the optoprobe is implanted in an animal’s brain, the body’s immune tissue treats the implanted device as a foreign object, causing rejection. The host response rejection causes astrocytes and microglia to aggregate and encapsulate the encapsulation probe [116,117], so that the electrodes could not record the action potentials of neurons. A drivable part which can lead the probe tip to break through the wrap and continue to function becomes a good choice. The drivable optoprobes are inspired by the micro-actuated structure of the electrodes [118,119], which can be customized according to the different optical components (Figure 3a,b) [97,116] or simpler 3D-printed molds [120,121,122]. The 3D-printed mold can also precisely control the adjustment step by twisting of the screw, shown in Figure 3c, and the minimum step is as low as 320 μm [122].

Moreover, three-dimensional optical probe arrays [123] allow measurements of neural activities across the whole region within an engineered 3D neural tissue, as well as measurements of the local modulations of the neural networks at a specific site [97]. Combining the 3D array with drivable structures assembled together is expected to enable long-term chronic recordings across different brain regions and depths (Figure 3d) [97,120,121].

#### 3.2.4. Flexible Optoprobes

Although the movable structure can penetrate the wrap, it does not fundamentally solve the rejection reaction. Inflammation resulting from the mismatch in Young’s modulus (~1–10 kPa) of traditional rigid photoelectrode implants (GPa) and brain tissue remains a major factor limiting the use of optical probes for chronic research and long-term implantation [89]. Flexible implantable devices have better biocompatibility and are more able to adapt to the deformation of nerve tissue caused by exercise [124], which can effectively prolong the working time in the body. Polymers such as Polydimethysiloxane (PDMS), polyimide (PI), Parylene C, silicone rubber, SU-8, and liquid crystal polymer are good choices for flexible substrates [89,107,108,125,126,127,128,129,130].

**Figure 4:**
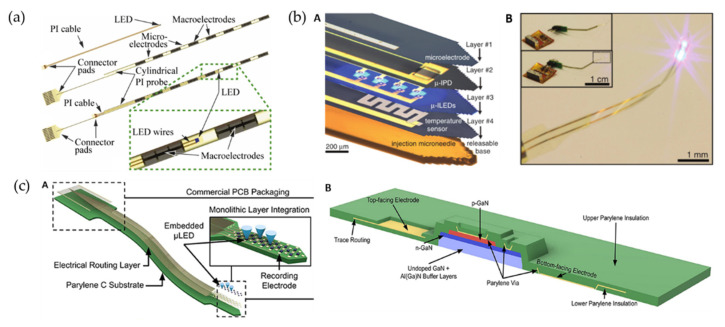
Several ways to implement flexible optoprobes: (**a**) Schematics of the cylindrical flexible optoprobe, in which a PI-based ribbon cable interconnects a bare LED chip by flip-chip bonding to respective connector pads. Close-up view showing the placement of the LED chip in a cylindrical transparent optoprobe between two macroelectrodes [132]. (**b**) Optoprobes design for transfer of printed μ-LED from sapphire wafer to a flexible polyester substrate: (**A**): Schematic diagram of its multi-layer structure; (**B**): Integrated system wirelessly powered with RF scavenging [134]. (**c**) Schematic illustration of the probe architecture with integrated GaN µ-LEDs and recording electrodes on a flexible substrate using a standard microfabrication process. (**A**): Schematic illustration of the probe architecture with a flexible Parylene C cable; (**B**): Schematic cross-section of active region [127]. Reprinted under a Creative Commons Attribution (CC BY) license.

Reddy et al. used PDMS as the substrate and Parylene C as the core of the waveguides to fabricate implantable waveguide arrays [131], which is a hopeful beginning of flexible waveguide optoprobes. However, integration of µ-LEDs on flexible substrates remains challenging. It is a feasible way to bond the LED chip onto the flexible probe shank by flip-chip bonding (Figure 4a) [132] or wire-bonding [133], but these complicated and cumbersome ways are not conducive to high-density integration. As illustrated in Figure 4b, Kim et al. transferred printed μ-LED from a sapphire wafer to a flexible polyester substrate, forming a wireless multifunctional optoprobe with a microelectrode layer, optical measurement-microscale inorganic photodetector (μ-IPD) layer, and a temperature sensor layer [134]. Reddy et al. monolithically integrated gallium nitride (GaN) µ-LEDs and recording electrodes on a flexible polymer substrate using a process that can be achieved in standard microfabrication facilities for the first time [127]. Specifically, they grew GaN-based heterostructures on a silicon wafer, monolithically integrated and encapsulated in a flexible polymer that includes interconnects, and released at the end of fabrication process. A schematic of the probe architecture is shown in Figure 4c.

## 4. Magnetrodes

### 4.1. Magnetophysiology

Magnetophysiology, which refers to the measurement of the magnetic field generated by ionic currents, has many advantages compared with electrophysiology. First of all, there is no need for direct contact with the field source during magnetic field measurement. Even if the sensor is wrapped due to rejection after long-term implantation, it can still detect brain magnetic signals as long as the insulation is reliable. In addition, the benefits of magnetic recording include: (1) The electric field strongly depends on the electrical conductivity of the tissue between the nerve cell and the recording electrode, and the EEG signal is affected by the large difference in electrical conductivity between different tissues. While the magnetic permeability of most biological tissues is uniform and basically the same as that in air, there is no distortion when propagating through the tissue, and it only decays with increasing distance from the current source; (2) The electrode measurement is always relative to the potential of the reference electrode, and the position and type of the reference electrode have a great influence on the measurement signal. In multi-electrode recordings, all channels usually share the same reference electrode, so the resulting signals are not independent, which also poses problems for the analysis of functional connectivity. On the other hand, magnetic recording requires no reference, so current signals can be measured directly without interference; (3) Electrical measurement records scalar values, while magnetic recording can set several sensors with different sensitive directions on the same probe to obtain vector information about the magnitude and direction of the current source, so it is expected to precisely locate the neuronal activity.

However, magnetophysiology is much less developed than electrophysiology, mainly because of the weakness of the magnetic field. Specifically, according to a simple model, the magnetic field generated by a single neuron is on the order of several pT. For the local magnetic field, assuming a certain number of neurons are regularly arranged in bundles and perfectly synchronized, then the magnetic field for 100 neurons is approximately 260 pT. These signals are typically seven to nine orders of magnitude lower than the Earth’s magnetic field; thus, they require very sensitive magnetic sensors to detect.

### 4.2. MR Sensors

Magnetoresistance (MR) sensors, whose resistance change as a function of the applied magnetic field, can realize reliable magnetic signal detection in the nT to pT range at room temperature using micron-sized structures [135,136,137]. MR sensor fabrication relies on a large-scale process, being compatible with Si-based integrated circuits which means low power consumption and cost, and makes it suitable for integration with different components [138,139,140,141,142,143]. The first type of MR sensor is an anisotropic magnetoresistance (AMR) sensor, which exploits the angle between the magnetic field and current [144]. More recently, spintronics, which takes advantage of the intrinsic property of electron spin to manipulate the macroscopic magnetism of materials, has opened the way to new MR sensors: the giant magnetoresistance (GMR) sensor and the tunnel magnetoresistance (TMR) sensor with higher sensitivity and smaller size. These two types of sensors based on ferromagnetic (FM)/non-magnetic (NM) heterostructures are what we mainly discuss here.

**Figure 5:**
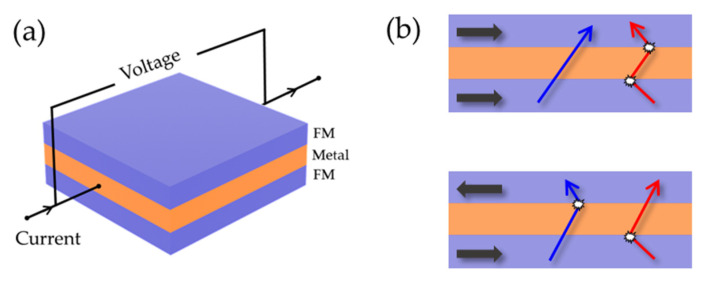
(**a**) Schematic representation of the GMR element, where current is flowing parallel to the film. (**b**) Schematic illustration of the two current models of GMR effect, explaining spin-dependent scattering of parallel magnetization and anti-parallel magnetization at the interface between FM and NM metal layers. Black arrows: the magnetization state of the FM layer. Blue arrows: trajectories of spin-up conduction electron. Red arrow: trajectories of spin-down conduction electron.

The GMR effect was independently discovered by Grunberg [145] and Fert [146] in 1988, and recognized with the Nobel Prize in 2007. It is caused by the asymmetry of spin-dependent scattering of spin-up and spin-down electrons at the FM/NM interface. In sensor applications, the spin-valve (SV) multilayer structure proposed by Dieny et al. [147] is widely used because of its better linearity [148,149] and the transport measurements are performed in a standard four-point probe with the current-in-plane (CIP) geometry (Figure 5a). The simplest SVs comprise two FM layers separated by a metal intermediate layer. The magnetization of one FM layer is free to rotate under application of a weak magnetic field while the other is pinned by coupling with an adjacent antiferromagnetic layer using the so-called exchange anisotropy phenomenon [150]. According to the two current models proposed by Mott [151], the phenomenological explanation of the GMR effect can be obtained. Figure 5b shows the movement of conduction electrons in a multilayer film sample under different applied magnetic fields. When the magnetization directions of the adjacent magnetic layers are distributed in parallel, two FM/NM interfaces show different resistance states. One interface is high-resistance state, while the other is low-resistance state; the spin conduction electrons can move freely, and the overall device presents a low-resistance state. When adjacent magnetic layers are distributed anti-parallel, both spin-up and spin-down conduction electrons will encounter a magnetic layer with the opposite spin direction, and are strongly scattered there. As a result, no electrons in any spin state can pass through the FM/NM interface, and the device presents a high-resistance state.

**Figure 6:**
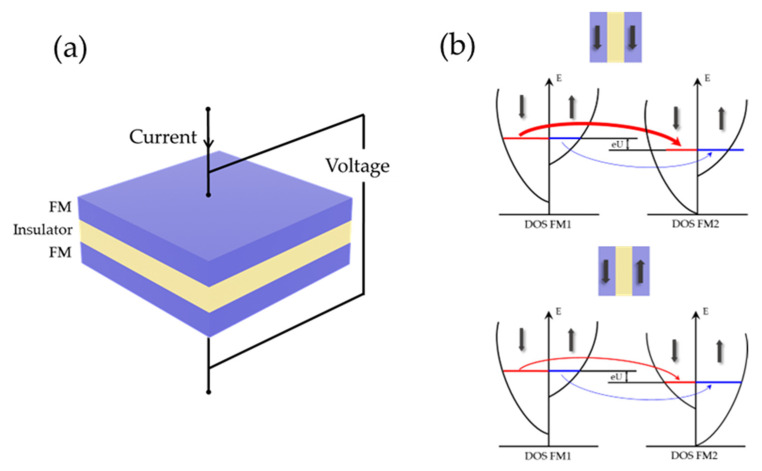
(**a**) Schematic representation of the TMR element, where current is flowing perpendicular to the film. (**b**) Spin subbands of parallel and anti-parallel magnetizations of FM materials. Black arrows: the magnetization state of the FM layer. Blue arrows: trajectories of spin-up conduction electron. Red arrow: trajectories of spin-down conduction electron. Thick (thin) arrows indicate high (low) spin currents.

Since then, scientists have been exploring the control of electron spin and thin film magnetic orientation at the atomic scale. Later, the discovery of MgO-barrier with thickness below 2 nm became a breakthrough in magnetic tunnel junction (MTJ)-based TMR sensors [152]. If the bias voltage is applied on the MTJ structure of FM layer/insulator layer/FM layer, the spin current can tunnel the insulator layer due to the spin tunneling effect [153], which seems impossible in classical physics. TMR originates from the energy level difference between spin-up electrons and spin-down electrons in density of states (DOS). During tunneling, electrons can only tunnel from a given spin subband in the first FM to the same spin subband in the second FM due to the conservation of spin orientation, as shown in Figure 6. The output exhibits low resistance when the magnetic moments of the two FM layers are arranged in parallel, and high resistance when they are arranged in an anti-parallel configuration. Therefore, the MTJ output can reflect the external magnetic field strength.

### 4.3. Magnetrodes

In view of the advantages of high sensitivity, miniaturization, and room temperature sensing, MR sensors have been designed as planar sensor arrays placed under the hippocampal brain slice to measure magnetic signals. Further, in order to achieve in vivo measurements and meanwhile minimize damage, “magnetrodes” have been proposed which are analogous to electrodes [154]. Integrated MR sensors onto a sharp silicon probe opens up the development of the magnetrodes.

**Figure 7:**
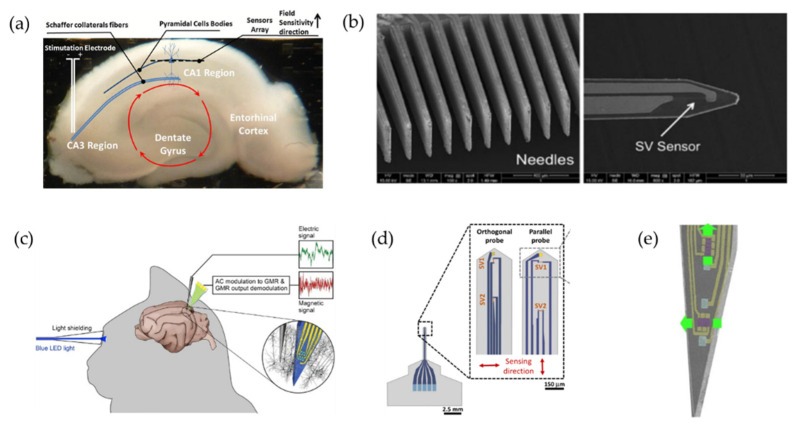
(**a**) Hippocampus slice with the relative position of the sensor array with respect to the hippocampus structure. Red arrows represent the hippocampal network forming a well-characterized closed loop due to synaptic and action potential sources [155]. Reprinted with permission from AIP Publishing 2011. (**b**) SEM image of the needles array and the tip of the needle with a well-defined SV sensor [157]. (**c**) Schematic representation of the in vivo experimental set-up to record neural responses from rat cerebral cortex [22]. Reprinted with permission from Elsevier 2017. (**d**) Schematic view of the sharp magnetrodes with orthogonal and parallel configurations and the position of SV1, SV2, and gold electrode along the probe. The arrows indicate the sensing direction [159]. (**e**) Photograph of the GMR magnetrode with orthogonally sensitive directions. Reprinted with permission from [161]. Copyright 2020 American Chemical Society.

The recording of neural activity by MR sensors was first attempted by Amaral et al. [155,156] in 2011, and follow-up works have been conducted since then. In their first work, a planar chip of 15 SV sensors prepared by microfabrication was placed under the CA1 region of the mouse hippocampal brain slice (Figure 7a). The hippocampus was selected because the pyramidal cells in the hippocampus are highly organized, which means that they are arranged in parallel and all cells are on the same plane, thus providing a larger superposition of magnetic fields. When electrical impulse stimulations were applied to the CA3 region, the resulting stimulation propagated along the fibers to the CA1 pyramidal neuron region, where it was detected by the underlying sensors. In order to verify the authenticity of this signal, they inhibited the action potential with tetrodotoxin (TTX), and the signal pulse stopped, confirming that the signal measured by the sensor originated from the action potential. However, the distance between the biological sources of the signal of interest and the sensor (about 10μm) constrains its further development. Two years later, they developed a hybrid device capable of being physically inserted within the brain slice that combines 15 silicon probes and MR sensors by placing the SV-GMR sensor (Figure 7b) [157] or MTJ-TMR sensor [158] at the end of each needle tip, which is the prototype of magnetrodes and is expected to measure of the magnetic field in different regions. However, in fact, they only measured a single sensor located in the area of pyramidal cells and observed the same type of signals as previous studies [155]. A common problem for both studies was that there was no comparison with other existing techniques for recording neural responses to verify the accuracy of the acquired signals.

Later in 2017, Caruso et al. [22] formally proposed the concept of “magnetrodes” and reported on in vivo magnetic recordings in the cat visual cortex with five segments of 4 × 30 µm^2^ SV-GMR arranged in a meander configuration. They averaged over multiple stimulus repetitions to calculate the event-related field (ERF), which was on the order of several nT. Although the SNR was still not satisfactory, this experiment shows the potential to exploit the fundamental advantages of magnetophysiology. In the same year, Valadeiro et al. [159] integrated two SV-GMR sensors (40 × 2 μm^2^) with a thin film gold electrode (20 × 20 μm^2^) at the tip of a single probe. They designed two different magnetrodes whose sensing direction was along the magnetrode length or width, and penetrated the hippocampal tissue with an inclination; the bending of the magnetrode’s tip will not affect the device performance [160], mainly for MR sensors. The gold electrode was responsible for verifying the biological signals and cell activities (live/dead) during the experiment. A recent study demonstrated a GMR magnetrode with two orthogonally sensitive directions, thanks to Joule heating-induced localized magnetization reorientation technique.

Another interesting part of this work is the research about biocompatibility. The multi-layer films of MR sensors contain elements such as Co, Fe, and Mn, which are toxic to organisms. On the other hand, interstitial fluid may also damage sensors on the magnetrodes. Therefore, it is essential to find a suitable passivation layer to ensure cell activity and protect the sensor. Sharma et al. [162] preliminarily studied the survival of neurons cultured for 2 weeks on MTJ arrays protected by SiO_2_(50)/Si_3_N_4_(70)/SiO_2_(50) (thickness in nm) trilayer which were all deposited by magnetron sputtering and showed about 50% cell viability. Moretti et al. [163] then conducted a more systematic study of the biocompatibility of magnetrodes. They reduced the thickness of the intermediate Si_3_N_4_ layer to 25 nm in order to bring the sensor closer to the field source, and found that the SiO_2_ layer was deposited by e-beam evaporation, while Si_3_N_4_ grown by magnetron sputtering had better compactness [164] and cell adhesion [165].

In earlier studies [155,156,157,158], a common problem was that the actual measured signal value was often bigger than the theoretical prediction [166], but still there is a lack of specific analysis of the reason/its causes. The first contribution came from Valadeiro et al. [159] who concluded that parasitic effects, generated from resistive, inductive, and capacitive couplings, may induce an electrical component when magnetrodes record the signal in the experiment. Then, Caruso et al. [22] developed a measurement scheme capable of suppressing capacitive coupling which was the main cause of interference signals. In this scheme, the sensors were powered by a high-frequency alternating current (AC) and their output was demodulated for both in-phase and out-of-phase components of AC modulation. As a result, magnetic signals were almost completely reflected in the in-phase component, while electrical signals were mainly reflected in the out-of-phase component.

## 5. Conclusions and Outlook

In this report, we provide the first comprehensive review of the latest research progress in flexible electrodes, as well as two novel neural probes—optoprobes and magnetrodes, and focused on their potential for applications in brain research and BCI. Flexible neural microelectrodes are more consistent with soft and dynamic neural tissue properties, and have outstanding advantages in biocompatibility, conductivity, and reliability. The use of bioresorbable coatings could help the flexible electrodes better insert into brain tissue. However, there are still some problems in clinical application, such as low interface matching which will affect signal quality and long-term compatibility. Therefore, exploring new advanced flexible materials and optimizing the manufacturing process will be the long-term theme of the next generation of flexible devices.

Optogenetics has opened new opportunities in the study of brain function, and optoprobes provide a strong support for optogenetics to achieve precise regulation. Depending on the way the optical components are integrated, three types of optical probes—optical fiber probes, MEMS waveguide integrated probes, and μ-LED-integrated probes—have been developed, and there are many studies that have been devoted to increasing the number of electrical recording and optical stimulation channels to improve their functionality. On this basis, preliminary studies of the various new structures of optoprobes, such as multifunctional optoprobes, artifact-free optoprobes, three-dimensional drivable optoprobes, and flexible optoprobes have shown overall feasibility, but much work remains to be done to make these methods long-term, stable, and reliable for long-term implantation. In addition to controlling spiking activity at the somatodendritic level, there has recently been growing interest in using optogenetic tools to understand synaptic plasticity [167] and further understand learning/memory [168,169]. In vitro [170,171,172] and in vivo [173,174,175,176] plasticity has been studied by different research groups. These optogenetic tools provide neuroscientists with a variety of experimental methods to finely manipulate neurons with unparalleled spatiotemporal resolution, which will facilitate the further development of neuroscience and BCI.

With regard to magnetrodes, application of neural recordings with MR sensors are still a long way off. At present, the research of magnetrodes mostly focuses on the structural design of the device; the detection of ultra-weak magnetic signals by a single MR sensor remains a challenging task, as the noise in the low-frequency region of the sensor so far is comparable to or even higher than the signal. In addition to reducing the intrinsic noise of the sensor by optimizing the MR stack structure and other means, connecting multiple sensors in series within a certain footprint is also a good choice for reducing low-frequency noise. Last but not least, there are still few in vivo experiments with magnetrodes and no standard operating procedure to regulate the acquisition of neural magnetic signals [177]. With the deepening of research, in addition to solving the above problems, the integration of MR sensors with flexible substrates to improve the biocompatibility of magnetrodes is also an encouraging development direction. Using magnetrodes to measure magnetic signals is an excellent choice to supplement existing electrophysiological techniques, and it is believed that it will get more and more attention and research.

Various new types of neural probes have been extensively studied by scientists all over the world, but from animals to humans, and from laboratories to markets, we still have a long way to go to improve the daily life of people who have lost the ability to move or speak. Many leading scientists are working with companies to solve technical problems such as damage to the brain caused by implanted devices, accuracy in reading input, and portability. Blackrock Neurotech has been working in this field for 18 years and currently has a variety of electrodes such as Utah array, Slant array, and NeuroPort array, and is still developing a fully implantable wireless BCI with less invasiveness. Neuralink’s system integrates it all into a tiny coin-sized device for wireless charging and transmission, but it needs to remove part of the skull and complete the implantation surgery by a robot. Interestingly, Synchron has developed a stent called “stentrode” with 16 electrodes molded around a vessel stent. The device, which enters the brain through blood vessels at the base of the neck, requires no drilling of the skull and no wires exiting the head or body, and is currently FDA-approved. However, such scaffolds have poor resolution and therefore cannot be used to control complex prosthetics [178]. As we all know, differences between people will bring great challenges to the practical application of BCI, so it is also crucial to develop more specific dedicated brain–computer interface chips in the future. In addition, one of the biggest difficulties is the controversy of safety, effectiveness, and the ethics of implanted technology; we need normative ethical oversight and the establishment of technology to prevent information leakage to protect users.

## Figures and Tables

**Table 1:** Comparison of three types of optical-stimulation-induced artifacts.

Type	Source	Magnitude	Suppression Methods
PV	Metal electrodes exposed to optical radiation	Tens to hundreds μVs	Heavily boron-doped silicon substrates [100]
			Transparent electrode materials [52,105]
			Flexible polymer-based substrates [106,107,108]
EMI	Active μ-LEDs and their interconnects	Several mVs	Metal shielding layer [100,108,110]
			Dual-metal-layer shielding [111]
			Transient pulse shaping [110]
PEC	Metal–electrolyte interface	Tens to hundreds μVs	Electrochemical modification [52,106,115]

## Data Availability

Not applicable.

## Abbreviations

AMR	anisotropic magnetoresistance
BCI	brain–computer interface
CNTs	carbon nanotubes
CPE	composite-conductive polyethylene
DOS	density of states
ERF	event-related field
FDA	Food and Drug Administration
GaN	gallium nitride
GRIN	gradient-index
MEG	magnetoencephalography
MR	magnetoresistive
NM	non-magnetic
PC	polycarbonate
PEC	photoelectrochemical
PEG	poly (ethylene glycol)
PI	polyimide
SNR	signal-to-noise ratio
TMR	tunneling magnetoresistance
UEA	Utah electrode array
AC	alternating current
CIP	current-in-plane
COC	cyclic olefin copolymer
CPs	conductive polymers
EEG	electroencephalogram
EMI	electromagnetic interference
FM	ferromagnetic
fNIRS	functional near-infrared spectroscopy
GMR	giant magnetoresistance
MEAs	microelectrode arrays
MEMS	microelectromechanical systems
MTJ	magnetic tunnel junction
nT/pT/fT	nano/pico/femto tesla
PDMS	polydimethylsiloxane
PEDOT	Poly (3,4-ethylenedioxythiophene)
PLGA	poly (lactic-co-glycolic acid)
PV	photovoltaic
SV	spin-valve
TTX	tetrodotoxin

## References

[1] Azevedo F.A.C., Carvalho L.R.B., Grinberg L.T., Farfel J.M., Ferretti R.E.L., Leite R.E.P., Jacob W., Lent R., Herculano-Houzel S. (2009). Equal Numbers of Neuronal and Nonneuronal Cells Make the Human Brain an Isometrically Scaled-Up Primate Brain. J. Comp. Neurol..

[2] Fetz E.E. (2015). Restoring motor function with bidirectional neural interfaces. Prog. Brain Res..

[3] Bell C.J., Shenoy P., Chalodhorn R., Rao R.P.N. (2008). Control of a humanoid robot by a noninvasive brain-computer interface in humans. J. Neural Eng..

[4] Kansaku K., Hata N., Takano K. (2010). My thoughts through a robot’s eyes: An augmented reality-brain-machine interface. Neurosci. Res..

[5] Vázquez-Guardado A., Yang Y., Bandodkar A.J., Rogers J.A. (2020). Recent advances in neurotechnologies with broad potential for neuroscience research. Nat. Neurosci..

[6] Millán Jdel R., Renkens F., Mouriño J., Gerstner W. (2004). Noninvasive brain-actuated control of a mobile robot by human EEG. IEEE Trans. Biomed. Eng..

[7] Rebsamen B., Guan C., Zhang H., Wang C., Teo C., Ang M.H., Burdet E. (2010). A brain controlled wheelchair to navigate in familiar environments. IEEE Trans. Neural Syst. Rehabil. Eng..

[8] Müller K.R., Tangermann M., Dornhege G., Krauledat M., Curio G., Blankertz B. (2008). Machine learning for real-time single-trial EEG-analysis: From brain-computer interfacing to mental state monitoring. J. Neurosci. Methods.

[9] Pfurtscheller G., da Silva F.H.L. (1999). Event-related EEG/MEG synchronization and desynchronization: Basic principles. Clin. Neurophysiol..

[10] Fazli S., Mehnert J., Steinbrink J., Curio G., Villringer A., Müller K.R., Blankertz B. (2012). Enhanced performance by a hybrid NIRS-EEG brain computer interface. Neuroimage.

[11] Coyle S.M., Ward T.E., Markham C.M. (2007). Brain-computer interface using a simplified functional near-infrared spectroscopy system. J. Neural Eng..

[12] Kauhanen L., Nykopp T., Lehtonen J., Jylänki P., Heikkonen J., Rantanen P., Alaranta H., Sams M. (2006). EEG and MEG brain-computer interface for tetraplegic patients. IEEE Trans. Neural Syst. Rehabil. Eng..

[13] Yeom H.G., Kim J.S., Chung C.K. (2013). Estimation of the velocity and trajectory of three-dimensional reaching movements from non-invasive magnetoencephalography signals. J. Neural Eng..

[14] Gross J. (2019). Magnetoencephalography in Cognitive Neuroscience: A Primer. Neuron.

[15] Hamalainen M., Hari R., Ilmoniemi R.J., Knuutila J., Lounasmaa O.V. (1993). Magnetoencephalography—Theory, instrumentation, and applications to noninvasive studies of the working human brain. Rev. Mod. Phys..

[16] Seymour J.P., Wu F., Wise K.D., Yoon E. (2017). State-of-the-art MEMS and microsystem tools for brain research. Microsyst. Nanoeng..

[17] Choi J.-R., Kim S.-M., Ryu R.-H., Kim S.-P., Sohn J.-W. (2018). Implantable Neural Probes for Brain-Machine Interfaces? Current Developments and Future Prospects. Exp. Neurobiol..

[18] Cogan S.F. (2008). Neural stimulation and recording electrodes. Annu. Rev. Biomed. Eng..

[19] Viswam V., Obien M.E.J., Franke F., Frey U., Hierlemann A. (2019). Optimal Electrode Size for Multi-Scale Extracellular-Potential Recording From Neuronal Assemblies. Front. Neurosci..

[20] Alt M.T., Fiedler E., Rudmann L., Ordonez J.S., Ruther P., Stieglitz T. (2017). Let There Be Light—Optoprobes for Neural Implants. Proc. IEEE.

[21] Boyden E.S., Zhang F., Bamberg E., Nagel G., Deisseroth K. (2005). Millisecond-timescale, genetically targeted optical control of neural activity. Nat. Neurosci..

[22] Caruso L., Wunderle T., Lewis C.M., Valadeiro J., Trauchessec V., Trejo Rosillo J., Amaral J.P., Ni J., Jendritza P., Fermon C. (2017). In Vivo Magnetic Recording of Neuronal Activity. Neuron.

[23] Chowdhury T. (1969). Fabrication of extremely fine glass micropipette electrodes. J. Phys. E Sci. Instrum..

[24] Simons D.J., Land P.W. (1987). A reliable technique for marking the location of extracellular recording sites using glass micropipettes. Neurosci. Lett..

[25] Pine J. (1980). Recording action potentials from cultured neurons with extracellular microcircuit electrodes. J. Neurosci. Methods.

[26] Palmer C. (1978). A microwire technique for recording single neurons in unrestrained animals. Brain Res. Bull..

[27] Lehew G., Nicolelis M.A.L. (2008). State-of-the-Art Microwire Array Design for Chronic Neural Recordings in Behaving Animals.

[28] Verloop A.J., Holsheimer J. (1984). A simple method for the construction of electrode arrays. J. Neurosci. Methods.

[29] Fekete Z. (2015). Recent advances in silicon-based neural microelectrodes and microsystems: A review. Sens. Actuators B Chem..

[30] Barrese J.C., Rao N., Paroo K., Triebwasser C., Vargas-Irwin C., Franquemont L., Donoghue J.P. (2013). Failure mode analysis of silicon-based intracortical microelectrode arrays in non-human primates. J. Neural Eng..

[31] Ghane-Motlagh B., Sawan M. (2013). Design and implementation challenges of microelectrode arrays: A review. Mater. Sci. Appl..

[32] Bretag A.H. (2017). The glass micropipette electrode: A history of its inventors and users to 1950. J. Gen. Physiol..

[33] Strumwasser F. (1958). Long-Term Recording from Single Neurons in Brain of Unrestrained Mammals. Science.

[34] Nicolelis M.A.L., Dimitrov D., Carmena J.M., Crist R., Lehew G., Kralik J.D., Wise S.P. (2003). Chronic, multisite, multielectrode recordings in macaque monkeys. Proc. Natl. Acad. Sci. USA.

[35] Jones K.E., Campbell P.K., Normann R.A. (1992). A glass/silicon composite intracortical electrode array. Ann. Biomed. Eng..

[36] Campbell P.K., Jones K.E., Huber R.J., Horch K.W., Normann R.A. (1991). A silicon-based, three-dimensional neural interface: Manufacturing processes for an intracortical electrode array. IEEE Trans. Biomed. Eng..

[37] Scholvin J., Kinney J.P., Bernstein J.G., Moore-Kochlacs C., Kopell N., Fonstad C.G., Boyden E.S. (2016). Close-Packed Silicon Microelectrodes for Scalable Spatially Oversampled Neural Recording. IEEE Trans. Biomed. Eng..

[38] Bhandari R., Negi S., Solzbacher F. (2010). Wafer-scale fabrication of penetrating neural microelectrode arrays. Biomed. Microdevices.

[39] Hochberg L.R., Serruya M.D., Friehs G.M., Mukand J.A., Saleh M., Caplan A.H., Branner A., Chen D., Penn R.D., Donoghue J.P. (2006). Neuronal ensemble control of prosthetic devices by a human with tetraplegia. Nature.

[40] Chaudhary U., Vlachos I., Zimmermann J.B., Espinosa A., Tonin A., Jaramillo-Gonzalez A., Khalili-Ardali M., Topka H., Lehmberg J., Friehs G.M. (2022). Spelling interface using intracortical signals in a completely locked-in patient enabled via auditory neurofeedback training. Nat. Commun..

[41] Kim S., Callier T., Tabot G.A., Gaunt R.A., Tenore F.V., Bensmaia S.J. (2015). Behavioral assessment of sensitivity to intracortical microstimulation of primate somatosensory cortex. Proc Natl Acad Sci USA.

[42] Flesher S.N., Downey J.E., Weiss J.M., Hughes C.L., Herrera A.J., Tyler-Kabara E.C., Boninger M.L., Collinger J.L., Gaunt R.A. (2021). A brain-computer interface that evokes tactile sensations improves robotic arm control. Science.

[43] Branner A., Normann R.A. (2000). A multielectrode array for intrafascicular recording and stimulation in sciatic nerve of cats. Brain Res. Bull..

[44] Bai Q., Wise K.D., Anderson D.J. (2000). A high-yield microassembly structure for three-dimensional microelectrode arrays. IEEE Trans. Biomed. Eng..

[45] Wise K.D., Angell J.B., Starr A. (1970). An integrated-circuit approach to extracellular microelectrodes. IEEE Trans. Biomed. Eng..

[46] Barz F., Livi A., Lanzilotto M., Maranesi M., Bonini L., Paul O., Ruther P. (2017). Versatile, modular 3D microelectrode arrays for neuronal ensemble recordings: From design to fabrication, assembly, and functional validation in non-human primates. J. Neural Eng..

[47] Wang X., Gu Y., Xiong Z., Cui Z., Zhang T. (2014). Silk-Molded Flexible, Ultrasensitive, and Highly Stable Electronic Skin for Monitoring Human Physiological Signals. Adv. Mater..

[48] Baek J.Y., An J.H., Choi J.M., Park K.S., Lee S.H. (2008). Flexible polymeric dry electrodes for the long-term monitoring of ECG. Sens. Actuators A Phys..

[49] Tang J., Guo H., Zhao M., Yang J., Tsoukalas D., Binzhen Z., Liu J., Xue C., Zhang W. (2015). Highly Stretchable Electrodes on Wrinkled Polydimethylsiloxane Substrates. Sci. Rep..

[50] Rousche P.J., Pellinen D.S., Pivin D.P., Williams J.C., Vetter R.J., Kipke D.R. (2001). Flexible polyimide-based intracortical electrode arrays with bioactive capability. IEEE Trans. Biomed. Eng..

[51] Moon J.H., Baek D.H., Choi Y.Y., Lee K.H., Kim H.C., Lee S.H. (2010). Wearable polyimide-PDMS electrodes for intrabody communication. J. Micromech. Microeng..

[52] Park D.-W., Schendel A.A., Mikael S., Brodnick S.K., Richner T.J., Ness J.P., Hayat M.R., Atry F., Frye S.T., Pashaie R. (2014). Graphene-based carbon-layered electrode array technology for neural imaging and optogenetic applications. Nat. Commun..

[53] Li W., Rodger D.C., Pinto A., Meng E., Weiland J.D., Humayun M.S., Tai Y.-C. (2011). Parylene-based integrated wireless single-channel neurostimulator. Sens. Actuators A Phys..

[54] Chen C.-H., Lin C.-T., Hsu W.-L., Chang Y.-C., Yeh S.-R., Li L.-J., Yao D.-J. (2013). A flexible hydrophilic-modified graphene microprobe for neural and cardiac recording. Nanomed. Nanotechnol. Biol. Med..

[55] Wu F., Im M., Yoon E. A flexible fish-bone-shaped neural probe strengthened by biodegradable silk coating for enhanced biocompatibility. Proceedings of the 2011 16th International Solid-State Sensors, Actuators and Microsystems Conference.

[56] Ansaldo A., Castagnola E., Maggiolini E., Fadiga L., Ricci D. (2011). Superior electrochemical performance of carbon nanotubes directly grown on sharp microelectrodes. ACS Nano.

[57] Sridharan A., Muthuswamy J. (2021). Soft, Conductive, Brain-Like, Coatings at Tips of Microelectrodes Improve Electrical Stability under Chronic, In Vivo Conditions. Micromachines.

[58] Shoval A., Adams C., David-Pur M., Shein M., Hanein Y., Sernagor E. (2009). Carbon nanotube electrodes for effective interfacing with retinal tissue. Front. Neuroeng..

[59] Hess L.H., Jansen M., Maybeck V., Hauf M.V., Seifert M., Stutzmann M., Sharp I.D., Offenhäusser A., Garrido J.A. (2011). Graphene transistor arrays for recording action potentials from electrogenic cells. Adv. Mater..

[60] Zhan B., Li C., Yang J., Jenkins G., Huang W., Dong X. (2014). Graphene field-effect transistor and its application for electronic sensing. Small.

[61] Du Z.-Z., Li W., Ai W., Tai Q., Xie L.-H., Cao Y., Liu J.-Q., Yi M.-D., Ling H.-F., Li Z.-H. (2013). Chemoselective reduction of graphene oxide and its application in nonvolatile organic transistor memory devices. RSC Adv..

[62] Green R., Abidian M.R. (2015). Conducting Polymers for Neural Prosthetic and Neural Interface Applications. Adv. Mater..

[63] Maziz A., Özgür E., Bergaud C., Uzun L. (2021). Progress in conducting polymers for biointerfacing and biorecognition applications. Sens. Actuators Rep..

[64] Kim D.H., Wiler J.A., Anderson D.J., Kipke D.R., Martin D.C. (2010). Conducting polymers on hydrogel-coated neural electrode provide sensitive neural recordings in auditory cortex. Acta Biomater..

[65] Luo X., Weaver C.L., Zhou D.D., Greenberg R., Cui X.T. (2011). Highly stable carbon nanotube doped poly(3,4-ethylenedioxythiophene) for chronic neural stimulation. Biomaterials.

[66] Vajrala V.S., Saunier V., Nowak L.G., Flahaut E., Bergaud C., Maziz A. (2021). Nanofibrous PEDOT-Carbon Composite on Flexible Probes for Soft Neural Interfacing. Front. Bioeng. Biotechnol..

[67] Sohal H.S., Clowry G.J., Jackson A., O’Neill A., Baker S.N. (2016). Mechanical Flexibility Reduces the Foreign Body Response to Long-Term Implanted Microelectrodes in Rabbit Cortex. PLoS ONE.

[68] Kee-Keun L., Jiping H., Amarjit S., Stephen M., Gholamreza E., Bruce K., Gregory R. (2004). Polyimide-based intracortical neural implant with improved structural stiffness. J. Micromech. Microeng..

[69] Felix S.H., Shah K.G., Tolosa V.M., Sheth H.J., Tooker A.C., Delima T.L., Jadhav S.P., Frank L.M., Pannu S.S. (2013). Insertion of flexible neural probes using rigid stiffeners attached with biodissolvable adhesive. J. Vis. Exp..

[70] Kim B.J., Kuo J.T., Hara S.A., Lee C.D., Yu L., Gutierrez C.A., Hoang T.Q., Pikov V., Meng E. (2013). 3D Parylene sheath neural probe for chronic recordings. J. Neural Eng..

[71] Kozai T.D., Kipke D.R. (2009). Insertion shuttle with carboxyl terminated self-assembled monolayer coatings for implanting flexible polymer neural probes in the brain. J. Neurosci. Methods.

[72] Takeuchi S., Ziegler D., Yoshida Y., Mabuchi K., Suzuki T. (2005). Parylene flexible neural probes integrated with microfluidic channels. Lab Chip.

[73] Foley C.P., Nishimura N., Neeves K.B., Schaffer C.B., Olbricht W.L. (2009). Flexible microfluidic devices supported by biodegradable insertion scaffolds for convection-enhanced neural drug delivery. Biomed. Microdev..

[74] Tien L.W., Wu F., Tang-Schomer M.D., Yoon E., Omenetto F.G., Kaplan D.L. (2013). Silk as a Multifunctional Biomaterial Substrate for Reduced Glial Scarring around Brain-Penetrating Electrodes. Adv. Funct. Mater..

[75] Jeon M., Cho J., Kim Y.K., Jung D., Yoon E.-S., Shin S., Cho I.-J. (2014). Partially flexible MEMS neural probe composed of polyimide and sucrose gel for reducing brain damage during and after implantation. J. Micromech. Microeng..

[76] Xiang Z., Yen S.-C., Xue N., Sun T., Tsang W.M., Zhang S., Liao L.-D., Thakor N.V., Lee C. (2014). Ultra-thin flexible polyimide neural probe embedded in a dissolvable maltose-coated microneedle. J. Micromech. Microeng..

[77] Kil D., Bovet Carmona M., Ceyssens F., Deprez M., Brancato L., Nuttin B., Balschun D., Puers R. (2019). Dextran as a Resorbable Coating Material for Flexible Neural Probes. Micromachines.

[78] Pas J., Rutz A.L., Quilichini P.P., Slézia A., Ghestem A., Kaszas A., Donahue M.J., Curto V.F., O’Connor R.P., Bernard C. (2018). A bilayered PVA/PLGA-bioresorbable shuttle to improve the implantation of flexible neural probes. J. Neural Eng..

[79] Lecomte A., Castagnola V., Descamps E., Dahan L., Blatché M.C., Dinis T.M., Leclerc E., Egles C., Bergaud C. (2015). Silk and PEG as means to stiffen a parylene probe for insertion in the brain: Toward a double time-scale tool for local drug delivery. J. Micromech. Microeng..

[80] Lecomte A., Descamps E., Bergaud C. (2018). A review on mechanical considerations for chronically-implanted neural probes. J. Neural Eng..

[81] Deisseroth K., Feng G.P., Majewska A.K., Miesenbock G., Ting A., Schnitzer M.J. (2006). Next-generation optical technologies for illuminating genetically targeted brain circuits. J. Neurosci..

[82] Tye K.M., Deisseroth K. (2012). Optogenetic investigation of neural circuits underlying brain disease in animal models. Nat. Rev. Neurosci..

[83] Nagel G., Szellas T., Huhn W., Kateriya S., Adeishvili N., Berthold P., Ollig D., Hegemann P., Bamberg E. (2003). Channelrhodopsin-2, a directly light-gated cation-selective membrane channel. Proc. Natl. Acad. Sci. USA.

[84] Zhang F., Wang L.-P., Boyden E.S., Deisseroth K. (2006). Channelrhodopsin-2 and optical control of excitable cells. Nat. Methods.

[85] Gradinaru V., Zhang F., Ramakrishnan C., Mattis J., Prakash R., Diester I., Goshen I., Thompson K.R., Deisseroth K. (2010). Molecular and Cellular Approaches for Diversifying and Extending Optogenetics. Cell.

[86] Gradinaru V., Thompson K.R., Zhang F., Mogri M., Kay K., Schneider M.B., Deisseroth K. (2007). Targeting and Readout Strategies for Fast Optical Neural Control In Vitro and In Vivo. J. Neurosci..

[87] Aravanis A.M., Wang L.P., Zhang F., Meltzer L.A., Mogri M.Z., Schneider M.B., Deisseroth K. (2007). An optical neural interface: In vivo control of rodent motor cortex with integrated fiberoptic and optogenetic technology. J. Neural Eng..

[88] Emara M.S., Pisanello M., Sileo L., de Vittorio M., Pisanello F. (2019). A Wireless Head-Mountable Device With Tapered Optical Fiber-Coupled Laser Diode for Light Delivery in Deep Brain Regions. IEEE Trans. Biomed. Eng..

[89] Sridharan A., Rajan S.D., Muthuswamy J. (2013). Long-term changes in the material properties of brain tissue at the implant–tissue interface. J. Neural Eng..

[90] Kampasi K., Stark E., Seymour J., Na K., Winful H.G., Buzsáki G., Wise K.D., Yoon E. (2016). Fiberless multicolor neural optoelectrode for in vivo circuit analysis. Sci. Rep..

[91] Kwon Y.W., Jun Y.S., Park Y.-G., Jang J., Park J.-U. (2021). Recent advances in electronic devices for monitoring and modulation of brain. Nano Res..

[92] Sharma K., Jäckel Z., Schneider A., Paul O., Diester I., Ruther P. (2021). Multifunctional optrode for opsin delivery, optical stimulation, and electrophysiological recordings in freely moving rats. J. Neural Eng..

[93] Park S., Guo Y., Jia X., Choe H.K., Grena B., Kang J., Park J., Lu C., Canales A., Chen R. (2017). One-step optogenetics with multifunctional flexible polymer fibers. Nat. Neurosci..

[94] Canales A., Jia X., Froriep U.P., Koppes R.A., Tringides C.M., Selvidge J., Lu C., Hou C., Wei L., Fink Y. (2015). Multifunctional fibers for simultaneous optical, electrical and chemical interrogation of neural circuits in vivo. Nat. Biotechnol..

[95] Rubehn B., Wolff S.B.E., Tovote P., Lüthi A., Stieglitz T. (2013). A polymer-based neural microimplant for optogenetic applications: Design and first in vivo study. Lab A Chip.

[96] Shin H., Son Y., Chae U., Kim J., Choi N., Lee H.J., Woo J., Cho Y., Yang S.H., Lee C.J. (2019). Multifunctional multi-shank neural probe for investigating and modulating long-range neural circuits in vivo. Nat. Commun..

[97] Shin H., Jeong S., Lee J.-H., Sun W., Choi N., Cho I.-J. (2021). 3D high-density microelectrode array with optical stimulation and drug delivery for investigating neural circuit dynamics. Nat. Commun..

[98] Sanja M., Stefano P., Helton Maia P., George C.D.N., Klas K., Adriano B.L.T., Richardson N.L. (2016). On the photovoltaic effect in local field potential recordings. Neurophotonics.

[99] Wang M., Fan Y., Li L., Wen F., Guo B., Jin M., Xu J., Zhou Y., Kang X., Ji B. (2022). Flexible Neural Probes with Optical Artifact-Suppressing Modification and Biofriendly Polypeptide Coating. Micromachines.

[100] Kim K., Vöröslakos M., Seymour J.P., Wise K.D., Buzsáki G., Yoon E. (2020). Artifact-free and high-temporal-resolution in vivo opto-electrophysiology with microLED optoelectrodes. Nat. Commun..

[101] Packer A.M., Roska B., Häusser M. (2013). Targeting neurons and photons for optogenetics. Nat. Neurosci..

[102] Ayling O.G.S., Harrison T.C., Boyd J.D., Goroshkov A., Murphy T.H. (2009). Automated light-based mapping of motor cortex by photoactivation of channelrhodopsin-2 transgenic mice. Nat. Methods.

[103] Han X., Qian X., Bernstein J.G., Zhou H.-H., Franzesi G.T., Stern P., Bronson R.T., Graybiel A.M., Desimone R., Boyden E.S. (2009). Millisecond-Timescale Optical Control of Neural Dynamics in the Nonhuman Primate Brain. Neuron.

[104] Cardin J.A., Carlén M., Meletis K., Knoblich U., Zhang F., Deisseroth K., Tsai L.-H., Moore C.I. (2010). Targeted optogenetic stimulation and recording of neurons in vivo using cell-type-specific expression of Channelrhodopsin-2. Nat. Protoc..

[105] Zatonyi A., Borhegyi Z., Srivastava M., Cserpan D., Somogyvari Z., Kisvarday Z., Fekete Z. (2018). Functional brain mapping using optical imaging of intrinsic signals and simultaneous high-resolution cortical electrophysiology with a flexible, transparent microelectrode array. Sens. Actuators B Chem..

[106] Guo B., Fan Y., Wang M., Cheng Y., Ji B., Chen Y., Wang G. (2021). Flexible Neural Probes with Electrochemical Modified Microelectrodes for Artifact-Free Optogenetic Applications. Int. J. Mol. Sci..

[107] Ji B., Ge C., Guo Z., Wang L., Wang M., Xie Z., Xu Y., Li H., Yang B., Wang X. (2020). Flexible and stretchable opto-electric neural interface for low-noise electrocorticogram recordings and neuromodulation in vivo. Biosens. Bioelectron..

[108] Guo Z.J., Ji B.W., Wang M.H., Ge C.F., Wang L.C., Gu X.W., Yang B., Wang X.L., Li C.Y., Liu J.Q. (2019). A Polyimide-Based Flexible Optoelectrodes for Low-Noise Neural Recording. IEEE Electron Device Lett..

[109] Kampasi K., English D.F., Seymour J., Stark E., McKenzie S., Vöröslakos M., Buzsáki G., Wise K.D., Yoon E. (2018). Dual color optogenetic control of neural populations using low-noise, multishank optoelectrodes. Microsyst. Nanoeng..

[110] Wang L.C., Ge C.F., Wang M.H., Ji B.W., Guo Z.J., Wang X.L., Yang B., Li C.Y., Liu J.Q. (2020). An artefact-resist optrode with internal shielding structure for low-noise neural modulation. J. Neural Eng..

[111] Kim K., English D., McKenzie S., Wu F., Stark E., Seymour J., Ku P.C., Wise K., Buzsaki G., Yoon E. GaN-on-Si μLED optoelectrodes for high-spatiotemporal-accuracy optogenetics in freely behaving animals. Proceedings of the 2016 IEEE International Electron Devices Meeting (IEDM).

[112] Laxpati N.G., Mahmoudi B., Gutekunst C.-A., Newman J.P., Zeller-Townson R., Gross R.E. (2014). Real-time in vivo optogenetic neuromodulation and multielectrode electrophysiologic recording with NeuroRighter. Front. Neuroeng..

[113] Budai D., Vizvári A.D., Bali Z.K., Márki B., Nagy L.V., Kónya Z., Madarász D., Henn-Mike N., Varga C., Hernádi I. (2018). A novel carbon tipped single micro-optrode for combined optogenetics and electrophysiology. PLoS ONE.

[114] Khurram A., Seymour J.P. (2013). Investigation of the photoelectrochemical effect in optoelectrodes and potential uses for implantable electrode characterization. Annu. Int. Conf. IEEE Eng. Med. Biol. Soc..

[115] Park J., Sun F., Xie Y., Xiong Z., Xu G. (2020). Low-Impedance Low-Artifact PEDOT: PSS-Coated Graphene Electrodes Towards High Density Optogenetic Electrophysiology. IEEE Electron Device Lett..

[116] Wang L., Huang K., Zhong C., Wang L., Lu Y. (2018). Fabrication and modification of implantable optrode arrays for in vivo optogenetic applications. Biophys. Rep..

[117] Polikov V.S., Tresco P.A., Reichert W.M. (2005). Response of brain tissue to chronically implanted neural electrodes. J. Neurosci. Methods.

[118] Lin L., Chen G., Xie K., Zaia K.A., Zhang S., Tsien J.Z. (2006). Large-scale neural ensemble recording in the brains of freely behaving mice. J. Neurosci. Methods.

[119] Wang M.-H., Ji B.-W., Gu X.-W., Guo Z.-J., Wang X.-L., Yang B., Li C.-Y., Liu J.-Q. (2018). A novel assembly method for 3-dimensional microelectrode array with micro-drive. Sens. Actuators B Chem..

[120] Wang M.H., Gu X.W., Ji B.W., Wang L.C., Guo Z.J., Yang B., Wang X.L., Li C.Y., Liu J.Q. (2019). Three-dimensional drivable optrode array for high-resolution neural stimulations and recordings in multiple brain regions. Biosens. Bioelectron..

[121] Wang L., Ge C., Wang F., Guo Z., Hong W., Jiang C., Ji B., Wang M., Li C., Sun B. (2021). Dense Packed Drivable Optrode Array for Precise Optical Stimulation and Neural Recording in Multiple-Brain Regions. ACS Sens..

[122] Stocke S.K., Samuelsen C.L. (2021). A drivable optrode for use in chronic electrophysiology and optogenetic experiments. J. Neurosci. Methods.

[123] Zorzos A.N., Scholvin J., Boyden E.S., Fonstad C.G. (2012). Three-dimensional multiwaveguide probe array for light delivery to distributed brain circuits. Opt. Lett..

[124] Zhao Z., Li X., He F., Wei X., Lin S., Xie C. (2019). Parallel, minimally-invasive implantation of ultra-flexible neural electrode arrays. J. Neural Eng..

[125] Hassler C., Boretius T., Stieglitz T. (2011). Polymers for neural implants. J. Polym. Sci. Part B Polym. Phys..

[126] Eickenscheidt M., Herrmann T., Weisshap M., Mittnacht A., Rudmann L., Zeck G., Stieglitz T. (2022). An optoelectronic neural interface approach for precise superposition of optical and electrical stimulation in flexible array structures. Biosens. Bioelectron..

[127] Reddy J.W., Kimukin I., Stewart L.T., Ahmed Z., Barth A.L., Towe E., Chamanzar M. (2019). High Density, Double-sided, Flexible Optoelectronic Neural Probes With Embedded mu LEDs. Front. Neurosci..

[128] Kohler F., Gkogkidis C.A., Bentler C., Wang X., Gierthmuehlen M., Fischer J., Stolle C., Reindl L.M., Rickert J., Stieglitz T. (2017). Closed-loop interaction with the cerebral cortex: A review of wireless implant technology. Brain-Comput. Interfaces.

[129] Gwon T.M., Kim C., Shin S., Park J.H., Kim J.H., Kim S.J. (2016). Liquid Crystal Polymer (LCP)-based Neural Prosthetic Devices. Biomed. Eng. Lett..

[130] Liu C., Zhao Y., Cai X., Xie Y., Wang T., Cheng D., Li L., Li R., Deng Y., Ding H. (2020). A wireless, implantable optoelectrochemical probe for optogenetic stimulation and dopamine detection. Microsyst. Nanoeng..

[131] Reddy J.W., Chamanzar M. (2018). Low-loss flexible Parylene photonic waveguides for optical implants. Opt. Lett..

[132] Schwaerzle M., Pothof F., Paul O., Ruther P. High-resolution neural depth probe with integrated 460 NM light emitting diode for optogenetic applications. Proceedings of the 2015 Transducers-2015 18th International Conference on Solid-State Sensors, Actuators and Microsystems (TRANSDUCERS).

[133] Ji B., Wang M., Kang X., Gu X., Li C., Yang B., Wang X., Liu J. (2017). Flexible Optoelectric Neural Interface Integrated Wire-Bonding *μ* LEDs and Microelectrocorticography for Optogenetics. IEEE Trans. Electron. Dev..

[134] Kim T.-I., McCall J.G., Jung Y.H., Huang X., Siuda E.R., Li Y., Song J., Song Y.M., Pao H.A., Kim R.-H. (2013). Injectable, Cellular-Scale Optoelectronics with Applications for Wireless Optogenetics. Science.

[135] Shen H.-M., Hu L., Fu X. (2018). Integrated Giant Magnetoresistance Technology for Approachable Weak Biomagnetic Signal Detections. Sensors.

[136] Wang M., Wang Y., Peng L., Ye C. (2019). Measurement of Triaxial Magnetocardiography Using High Sensitivity Tunnel Magnetoresistance Sensor. IEEE Sens. J..

[137] Gaster R.S., Hall D.A., Nielsen C.H., Osterfeld S.J., Yu H., Mach K.E., Wilson R.J., Murmann B., Liao J.C., Gambhir S.S. (2009). Matrix-insensitive protein assays push the limits of biosensors in medicine. Nat. Med..

[138] Cardoso S., Leitao D.C., Dias T.M., Valadeiro J., Silva M.D., Chicharo A., Silverio V., Gaspar J., Freitas P.P. (2017). Challenges and trends in magnetic sensor integration with microfluidics for biomedical applications. J. Phys. D Appl. Phys..

[139] Graham D.L., Ferreira H.A., Freitas P.P. (2004). Magnetoresistive-based biosensors and biochips. Trends Biotechnol..

[140] Gaster R.S., Xu L., Han S.-J., Wilson R.J., Hall D.A., Osterfeld S.J., Yu H., Wang S.X. (2011). Quantification of protein interactions and solution transport using high-density GMR sensor arrays. Nat. Nanotechnol..

[141] Li G., Sun S., Wilson R.J., White R.L., Pourmand N., Wang S.X. (2006). Spin valve sensors for ultrasensitive detection of superparamagnetic nanoparticles for biological applications. Sens. Actuators A Phys..

[142] Germano J., Martins V.C., Cardoso F.A., Almeida T.M., Sousa L., Freitas P.P., Piedade M.S. (2009). A portable and autonomous magnetic detection platform for biosensing. Sensors.

[143] Martins V.C., Cardoso F.A., Germano J., Cardoso S., Sousa L., Piedade M., Freitas P.P., Fonseca L.P. (2009). Femtomolar limit of detection with a magnetoresistive biochip. Biosens. Bioelectron..

[144] Schulz L., Heinisch P., Richter I. (2019). Calibration of Off-the-Shelf Anisotropic Magnetoresistance Magnetometers. Sensors.

[145] Binasch G., Grünberg P., Saurenbach F., Zinn W. (1989). Enhanced magnetoresistance in layered magnetic structures with antiferromagnetic interlayer exchange. Phys. Rev. B.

[146] Baibich M.N., Broto J.M., Fert A., Van Dau F.N., Petroff F., Etienne P., Creuzet G., Friederich A., Chazelas J. (1988). Giant Magnetoresistance of (001)Fe/(001)Cr Magnetic Superlattices. Phys. Rev. Lett..

[147] Dieny B., Speriosu V.S., Parkin S.S.P., Gurney B.A., Wilhoit D.R., Mauri D. (1991). Giant magnetoresistive in soft ferromagnetic multilayers. Phys. Rev. B.

[148] Heim D.E., Fontana R.E., Tsang C., Speriosu V.S., Gurney B.A., Williams M.L. (1994). Design and operation of spin valve sensors. IEEE Trans. Magn..

[149] Freitas P.P., Leal J.L., Melo L.V., Oliveira N.J., Rodrigues L., Sousa A.T. (1994). Spin-valve sensors exchange-biased by ultrathin TbCo films. Appl. Phys. Lett..

[150] Dieny B., Johnson M. (2004). Chapter 2—Spin valves. Magnetoelectronics.

[151] Wilson A.H., Fowler R.H. (1938). The electrical conductivity of the transition metals. Proc. R. Soc. Lond. Ser. A Math. Phys. Sci..

[152] Yuasa S., Djayaprawira D. (2007). Giant tunnel magnetoresistance in magnetic tunnel junctions with a crystalline MgO (0 0 1) barrier. J. Phys. D Appl. Phys..

[153] Julliere M. (1975). Tunneling between ferromagnetic films. Phys. Lett. A.

[154] Fermon C., Van de Voorde M. (2016). Nanomagnetism: Applications and Perspectives.

[155] Amaral J., Cardoso S., Freitas P.P., Sebastião A.M. (2011). Toward a system to measure action potential on mice brain slices with local magnetoresistive probes. J. Appl. Phys..

[156] Freitas P., Cardoso F., Martins V., Martins S., Loureiro J., Amaral J., Chaves R., Cardoso S., Fonseca L., Sebastião A. (2012). Spintronic platforms for biomedical applications. Lab A Chip.

[157] Amaral J., Gaspar J., Pinto V., Costa T., Sousa N., Cardoso S., Freitas P. (2013). Measuring brain activity with magnetoresistive sensors integrated in micromachined probe needles. Appl. Phys. A.

[158] Amaral J., Pinto V., Costa T., Gaspar J., Ferreira R., Paz E., Cardoso S., Freitas P.P. (2013). Integration of TMR Sensors in Silicon Microneedles for Magnetic Measurements of Neurons. IEEE Trans. Magn..

[159] Valadeiro J., Silva M., Cardoso S., Martins M., Gaspar J., Freitas P.P., Sebastião A.M. Microneedles with integrated magnetoresistive sensors: A precision tool in biomedical instrumentation. Proceedings of the 2017 IEEE Sensors Applications Symposium (SAS).

[160] Valadeiro J., Amaral J., Leitao D.C., Silva A.V., Gaspar J., Silva M., Costa M., Martins M., Franco F., Fonseca H. (2015). Bending Effect on Magnetoresistive Silicon Probes. IEEE Trans. Magn..

[161] Chopin C., Torrejon J., Solignac A., Fermon C., Pannetier-Lecoeur M. (2020). Magnetoresistive Sensor in Two-Dimension on a 25 μm Thick Silicon Substrate for In Vivo Neuronal Measurements. ACS Sens..

[162] Sharma P.P., Gervasoni G., Albisetti E., D’Ercoli F., Monticelli M., Moretti D., Forte N., Rocchi A., Ferrari G., Baldelli P. (2017). Towards a magnetoresistive platform for neural signal recording. AIP Adv..

[163] Moretti D., DiFrancesco M.L., Sharma P.P., Dante S., Albisetti E., Monticelli M., Bertacco R., Petti D., Baldelli P., Benfenati F. (2018). Biocompatibility of a Magnetic Tunnel Junction Sensor Array for the Detection of Neuronal Signals in Culture. Front. Neurosci..

[164] Vanhove E., Tsopéla A., Bouscayrol L., Desmoulin A., Launay J., Temple-Boyer P. (2013). Final capping passivation layers for long-life microsensors in real fluids. Sens. Actuators B Chem..

[165] Monticelli M., Conca D.V., Albisetti E., Torti A., Sharma P.P., Kidiyoor G., Barozzi S., Parazzoli D., Ciarletta P., Lupi M. (2016). Magnetic domain wall tweezers: A new tool for mechanobiology studies on individual target cells. Lab A Chip.

[166] Okada Y.C., Wu J., Kyuhou S. (1997). Genesis of MEG signals in a mammalian CNS structure. Electroencephalogr. Clin. Neurophysiol..

[167] Xie Y.F., Jackson M.F., Macdonald J.F. (2013). Optogenetics and synaptic plasticity. Acta Pharm. Sin..

[168] Huff M.L., Miller R.L., Deisseroth K., Moorman D.E., LaLumiere R.T. (2013). Posttraining optogenetic manipulations of basolateral amygdala activity modulate consolidation of inhibitory avoidance memory in rats. Proc. Natl. Acad. Sci. USA.

[169] Rolls A., Colas D., Adamantidis A., Carter M., Lanre-Amos T., Heller H.C., de Lecea L. (2011). Optogenetic disruption of sleep continuity impairs memory consolidation. Proc. Natl. Acad. Sci. USA.

[170] Xiong W., Jin X. (2012). Optogenetic field potential recording in cortical slices. J. Neurosci. Methods.

[171] Kohl M.M., Shipton O.A., Deacon R.M., Rawlins J.N., Deisseroth K., Paulsen O. (2011). Hemisphere-specific optogenetic stimulation reveals left-right asymmetry of hippocampal plasticity. Nat. Neurosci..

[172] Chun S., Bayazitov I.T., Blundon J.A., Zakharenko S.S. (2013). Thalamocortical long-term potentiation becomes gated after the early critical period in the auditory cortex. J. Neurosci..

[173] Shibata A.C.E., Ueda H.H., Eto K., Onda M., Sato A., Ohba T., Nabekura J., Murakoshi H. (2021). Photoactivatable CaMKII induces synaptic plasticity in single synapses. Nat. Commun..

[174] Liewald J.F., Brauner M., Stephens G.J., Bouhours M., Schultheis C., Zhen M., Gottschalk A. (2008). Optogenetic analysis of synaptic function. Nat. Methods.

[175] Adamantidis A.R., Tsai H.C., Boutrel B., Zhang F., Stuber G.D., Budygin E.A., Touriño C., Bonci A., Deisseroth K., de Lecea L. (2011). Optogenetic interrogation of dopaminergic modulation of the multiple phases of reward-seeking behavior. J. Neurosci..

[176] Sasaki T., Beppu K., Tanaka K.F., Fukazawa Y., Shigemoto R., Matsui K. (2012). Application of an optogenetic byway for perturbing neuronal activity via glial photostimulation. Proc. Natl. Acad. Sci. USA.

[177] Su D., Wu K., Saha R., Peng C., Wang J.-P. (2019). Advances in Magnetoresistive Biosensors. Micromachines.

[178] Drew L. (2022). The brain-reading devices helping paralysed people to move, talk and touch. Nature.

